# Impairment of the cell-to-matrix adhesion and cytotoxicity induced by the Mediterranean jellyfish Pelagia noctiluca venom and its fractions in cultured glioblastoma cells

**DOI:** 10.1186/1476-511X-11-84

**Published:** 2012-06-28

**Authors:** Yosra Ayed, Manel Bousabbeh, Hazem Ben Mabrouk, Maram Morjen, Naziha Marrakchi, Hassen Bacha

**Affiliations:** 1 Laboratory for Research on Biologically Compatible Compounds, Faculty of Dentistry, Rue Avicenne, Monastir, 5019, Tunisia; 2 Laboratoire des Venins et Toxines, Institut Pasteur de Tunis, 13 Place Pasteur, BP.74, 1002, Tunis Belvédère, Tunisia; 3 University of Jendouba, Cité AlFaeiz rue Jamil Boutheina, Jendouba, 8100, Tunisia; 4 Head of the Laboratory for Research on Biologically Compatible Compounds (LRSBC), Faculty of Dentistry, Monastir university, Rue Avicenne, Monastir, 5019, Tunisia

**Keywords:** *Pelagia noctiluca*, Venom, Sephadex G-75, Cell proliferation, Cell adhesion

## Abstract

**Background:**

The biodiversity of the marine environment and the associated chemical diversity constitute a practically unlimited source of new active substances in the field of the development of bioactive products. In our study, we have investigated the efficiency of the venom from the Mediterranean jellyfish, *Pelagia noctiluca* and its fractions for anti-proliferative and anti-cell adhesion to cell–extracellular matrix activities.

**Results:**

Our experiments have indicated that the separation of the Mediterranean jellyfish *Pelagia noctiluca* crude venom extract by sephadex G-75 chromatography led to four fractions (F1, F2, F3, and F4). Among the four fractions F1 and F3 were cytotoxic against U87 cells with IC50 values of 125 and 179 μg/ml respectively. The venom, F1, F2 and F 3 showed significant anti-proliferative activity in time-dependent manner. Our results also suggest that these fractions and the venom are able to inhibit cell adhesion to fibrinogen in dose-dependent manner. This inhibition is reliant on its ability to interact with integrins.

**Conclusions:**

To conclude, we have demonstrated for the first time that *Pelagia noctiluca* venom and its fractions especially (F1 and F2) display potent anti-tumoral properties. Separation by sephadex G-75 chromatography give rise to more active fractions than the crude venom extract. The purification and the determination of chemical structures of compounds of these active fractions are under investigation. Overall, *Pelagia noctiluca* venom may has the potential to serve as a template for future anticancer-drug development.

## Background

Gliomas are the most common form of brain tumour [1]. Malignant gliomas are highly invasive, and as such, are neurologically destructive. Survival rates are poor because present therapies, including surgery, radiation therapy and chemotherapy, have limited effectiveness [2]. Finding new therapeutic strategies targeting multiple cell signaling pathways, such as inhibition of proliferation, adhesion, migration and angiogenesis, have been a major point of concern for research in the pharmaceutical sciences.

Extensive research has shown that cell-adhesion activities are deregulated in many diseases such as cancer. Therefore, the characterization of molecules capable to inhibit these alterations is considered very important for the development of alternative therapies [3]. Snake venoms are complex mixtures of molecules possessing various biological functions. Researches have been shown that several snake venom-derived peptides can affect cell adhesion, proliferation and angiogenesis by interacting with integrins [3,4].

Recently, the marine environment has been recognized as a rich source of bioactive metabolites with varied biological and pharmacological activities [5-11]. Some anticancer agents were derived from the venoms of cnidarians. The most studied cnidarian compounds are the secondary metabolites from soft corals such as diterpenes, sesquiterpenes, terpenoids, and monoterpenoids [12]. The jellyfish *Pelagia noctiluca* (*P. noctiluca*) [13], (class Scyphozoa, order Semaestomeae, family Pelagiidae), is widely distributed in different parts of the Mediterranean Sea [14-16] and in the Atlantic Ocean [17]. Therefore, it is the most dangerous autochthonous Mediterranean jellyfish [16], able to reconstitute its stinging battery within few days after discharge [18]. Nematocysts (or cnidocysts) are characteristically used by jellyfish to defend themselves against predator and to capture prey. These organelles contain irritating poisons, and are armed with sharp hollow threads that deliver venom causing painful stings. Nematocysts are located along the tentacles and body. Proteins isolated from jellyfish, with a unique structure, have many bioactivities such as enzymatic activities, hemolysin, dermonecrotic factor, and cytolysin [16-19]. However, little is known about the anti-tumor activity of the active components of *P. noctiluca* venom and their mechanism of action on cancer cells remains unknown.

Thus, this study attempts, for the first time to characterize and evaluate the exact role of *P. noctiluca* venom and its semi-purified fractions on (i) viability of human glioblastoma cells (U87), (ii) cell proliferation and (iii) cell adhesion to immobilized extracellular matrix (ECM) protein (fibrinogen).

## Materials and methods

### Chemicals

3–4, 5-dimethylthiazol-2-yl, 2,5-diphenyltetrazolium bromide (MTT), Cell culture medium (RPMI1640), foetal calf serum (FCS), phosphate buffer saline (PBS), trypsin–EDTA, penicillin and streptomycin mixture and l-glutamine (200 mM) were from GIBCO-BCL (UK). Poly-L-lysine, Human fibrinogen was purchased from Sigma (St Quentin Fallavier, France). All other chemicals used were of analytical grade.

### Preparation of nematocysts

Specimens of *P. noctiluca* were collected from the bay of Monastir, Tunisia, in May 2011, and identified by professor Mohamed Nejib Daly Yahia from Faculty of Sciences of Bizerte, (Bizerte, Tunisia). Tentacles were excised manually from living specimens immediately after capture.

The nematocysts isolation method has been previously described by Arillo et al. [20] with a slight modification. Tentacles were submerged in distilled water for 5 h at 4°C. The ratio of organic tissue to distilled water was approximately 1:5 (v/v). After a complete detachment of the epidermis, the tissue was removed from the suspension containing both epidermis and undischarged nematocysts deriving from the osmotic rupture of nematocysts. The nematocysts, attached to the epidermal tissue, were separated by stirring. The nematocysts suspension was repeatedly washed in distilled water and filtered through plankton nets to remove most of the tissue debris, and then centrifuged at 4°C (ALC PK 120R, 4000 g for 5 min). The content, purity and integrity of nematocysts (cnidocysts) were controlled microscopically [21].

### Nematocysts lysis and protein extraction

Crude venom was extracted by sonication on ice (Sonoplus, 70 mHz, 30 times, 20 s) of nematocysts as described by Marino et al. [21]. After sonication, the suspension was centrifuged at 15,000 rpm for 15 min at 4°C. The supernatant was carefully removed, filtered and lyophilized.

### Protein determination

The protein content of *P. noctiluca* venom was determined according to the Bradford method (BioRad Labs, Hercules, CA) [22]. “venom and fractions concentrations” refer to protein concentration expressed in units of μg ml^-1^.

### Size exclusion chromatography

About 300 mg of crude venom of *P. noctiluca* was dissolved in filtered–degassed double-distilled water. After centrifugation at 17 000 g for 15 min at 4°C, the supernatant was loaded on Sephadex G-75 gel-filtration chromatography columns (2.6 × 100 cm; Pharmacia), previously equilibrated with 200 mM ammonium acetate, pH 6.8 and eluted under the same conditions. The flow rate was 3 ml/min using a Bio-Rad 2110 fraction collector and the elution of the proteins was monitored at 280 nm by an ultraviolet detector.

### Cell viability assay

Cytotoxicity of *P. noctiluca* crude venom and its fractions was defined using the colorimetric method described by Mossmann, [16]. The MTT test assesses cell metabolism based on the ability of the mitochondrial succinate-dehydrogenase to convert the yellow compound MTT to a blue formazan dye. The amount of dye produced is proportional to the number of live metabolically active cells.

Cells were seeded on 96-well culture plates (Polylabo, France) at 10^5^ cells/ well and treated with increasing concentrations of crude venom extract at 37°C. After 24 h, the culture medium was replaced by 200 μl medium containing 0.5 mg/ml MTT and the plates were incubated 3 hours at 37°C. The medium was then removed and replaced by 200 μl of (0,04 M HCl/isopropanol) to solubilize the converted purple dye in culture plates. The absorbance was measured on a spectrophotometer microplate reader (Dynatech 4000) at 560 nm.

Cell viability was expressed as the relative formazan formation in treated samples as compared to control cells (untreated cells) [(A560 treated cells/A560 control cells) 100%]. IC50 values are defined as the concentration that induces 50% loss of cell viability.

For morphological analysis, U87 cells were treated with 50 and 100 μg/ml of *P. noctiluca* crude venom or F1 or F2 or F3 or F4. Morphological changes were examined and recorded under an inverted phase contrast microscope (Olympus IX50 with a PMC35Dx photo micrograph system). Each experiment was performed in triplicates.

### Cell proliferation assay

U87 cells were plated (3 × 10^3^/well) in 96-well plates (Nunc, Denmark) in their complete medium and were incubated for 24 h before addition of *P. noctiluca* venom and its fractions. After the incubation, the normal medium was replaced for an extract containing medium at the concentrations of 10 μg/ml and treated for 48, 72, 96, and 120 h. The control cells were maintained in normal medium.

The effect of tested compounds on proliferation of U87 cell line was determined using the MTT assay (previously described). Absorbance of the colored solution was measured on a microplate photometer (Dynatech 4000) using a wavelength of 560 nm. The anti-proliferative effect of the tested extracts was determined by comparing the optical density of the treated cells against the optical density of the control (untreated cells).

### Cell adhesion assay

The human glioblastoma (U87) cell line was routinely cultured in RPMI (GIBCO) medium with 10% FCS. Adhesion assay was performed as previously described [23]. Briefly, flat bottom 96-well microtiter plates were coated with one of the following purified ECM proteins: 50 μg/ml fibrinogen (Fg) or poly-L-lysine (Poly-L) and were blocked with BSA. Cells were harvested in single cell suspension and resuspended in RPMI containing 0.2% BSA (adhesion buffer) in the presence or absence of *P. noctiluca* crude venom and its fractions. After incubation for 30 min at room temperature, cells were added to coated wells in a volume of 50 μl (10^6^ cells/ml) and allowed to adhere with the substrate for 1 h at 37°C in order to activate alpha v beta 3 integrin. Unattached cells were removed by gently washing three times with adhesion buffer. Residual attached cells were fixed by 1% glutaraldehyde, stained by 0.1% crystal violet and lysed with 1% SDS. Absorbance was then measured at 600 nm by a microplate reader.

### Statistical analysis

All data are expressed as means ± standard error of the mean (SEM) of at least 3 independent experiments. Statistical differences were evaluated by 1-way ANOVA followed by Tukey’s test using commercially available software (SPSS 17.0; SPSS Inc., Chicago, Ill.). P values <0.05 were considered statistically significant.

## Results

### Sephadex G-75 chromatography

Separation of crude venom of *P. noctiluca* was achieved by a size exclusion chromatography (sephadex G 75). This gel is a dextran capable of separating proteins with molecular weights between 3 and 70 KDa. Proteins with molecular weights greater than 70 KDa are completely excluded.

**Figure 1:**
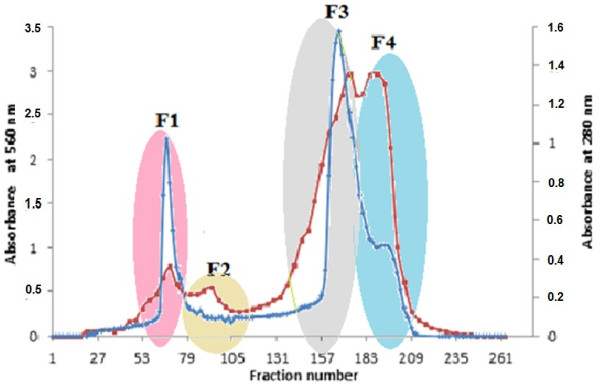
**Gel filtration chromatography of *P. noctiluca *crude venom on a Sephadex G75 column.** Elution was performed at 18 ml/h and 3 ml aliquots were collected. Four fractions and two major peaks were obtained.

The crude *P. noctiluca* venom (300 mg) was loaded on a previously equilibrated Sephadex G-75 column (2.6 × 100 cm; Pharmacia) after washing the column with 900 ml of 200 mM ammonium acetate buffer, pH 6.8. The venom components were eluted with the same buffer at a flow rate of 18 ml/h. Fractions were collected. Protein elution was monitored at 280 nm and protein contents were determined using the Bradford Method [22]. The fractions obtained were grouped into four groups and were pooled, lyophilized and evaluated in U87 cell line (Figure 1).

### Inhibition of cell viability

**Figure 2:**
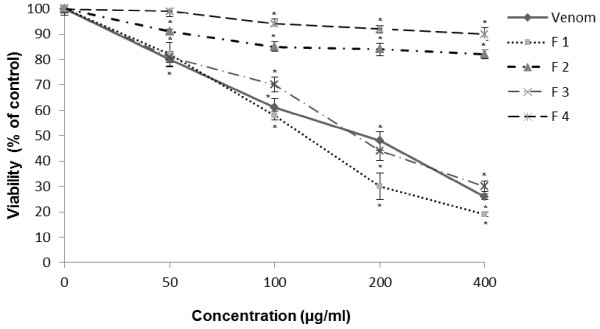
**Cytotoxic effect of *P. noctiluca *crude venom and fractions (F1 –F4) on U87 cells.** Cells were treated with venom and its fractions at the indicated concentrations for 24 h. Cell viability was determined using the MTT assay and expressed as percentages of control which was exposed to vehicle only. Control value was taken as 100%. Data are expressed as the mean ± S.E. *p < 0.05 compared with control (untreated cells).

The effect of crude venom of *P. noctiluca* and its fractions (from the gel filtration chromatography) on cells viability was assessed by the MTT assay [24]. Cultured cells were exposed to increasing concentrations of crude venom of *P. noctiluca* and its fractions for 24 h. As shown in Figure 1, all concentrations of crude venom (from 50 to 400 μg/ml) were sufficient to inhibit viability of U87 cells, with a dose-dependent characteristic (Figure 2). IC50 value was about 180 μg/ml (Table 1). However, we observed that all semi-purified fractions act differently on cell viability. Reduction in the viability of U87 cells by F1 has been already significant at low concentrations and the estimated IC50 was about 125 μg/ml. It indicates that F1 is more potent than the crude venom extract. A decrease of cell viability was also observed when cells were treated with F3, IC50 value was around 179 μg/ml (Table 1). F2 and F4 had no cytotoxic effect on U87 cells.

**Table 1:** **IC50 values of of *P. noctiluca *venom and its fractions (F1- F4) on U87 cell line as determined by cell viability and cell adhesion assays**

	Cell Viability	Cell Proliferation	Cell Adhesion
	IC_50_(μg/ml)		IC_50_(μg/ml)
Venom	180	++	25
F1	125	+++	5
F2	-	+++	64
F3	179	+	100
F4	-	-	-

Effects of *P. noctiluca* venom and its fractions (F1- F4) on cell proliferation, (−) no effect, (+) low, (++) medium, (+++) important.

**Figure 3:**
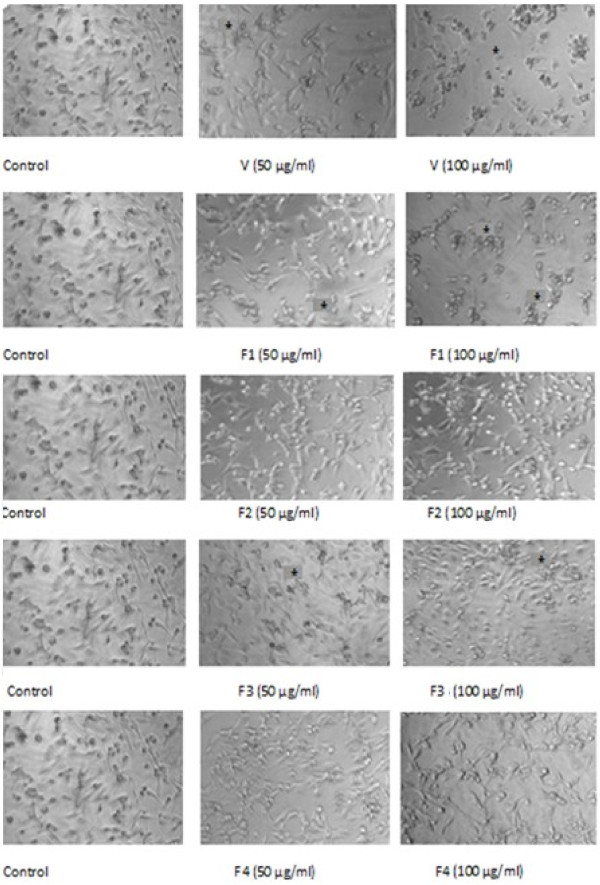
**Effects of 50 and 100 μg/ml of *P. noctiluca *venom or its fractions (F1-F4) on U87 cell morphology *in vitro***. This figure demonstrates that venom, F1 and F3 reduced cell growth when compared with untreated cells. Some cells displayed altered morphology (am). Cells were observed with phase contrast microscope (original magnification × 100).

The morphological and growth characteristics were assessed by observation of the cultures using an inverted phase contrast microscope. Cells treated with crude venom, F1 and F3 exhibited changes in their morphology (Figure 3 ()). During this incubation, a number of cells was also detached from the culture flasks and died.

### Inhibition of cell proliferation

U87 cells were grown in 96-well assay plates and their proliferation was measured over 5 days in four separate independent experimental trials to determine if the administration of *P. noctiluca* venom and its fractions at 10 μg/ml were sufficient to inhibit cellular proliferation. Cells did not receive fresh medium during these periods of incubation.

Our results demonstrated that comparison of treated cells to untreated control cells at day 2 to day 5 revealed that the venom, F1 and F2 had significant effect on cell proliferation when applied at 10 μg/ml (P < 0.05, Figure 1). 120 h of incubation with venom or its fractions were employed for U87 to demonstrate the extreme cytotoxic effect of *P. noctiluca* venom and its fractions on these cells. Despite the long period of incubation (120 h) without adding fresh medium, untreated U87 cells survived as well as those cells from a shorter period of incubation.

**Figure 4:**
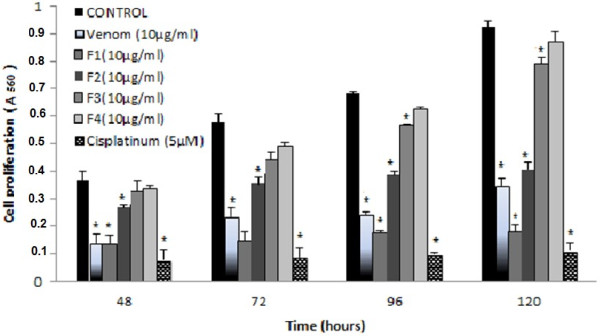
***P. noctiluca *venom or its fractions (F1-F4) inhibit tumour cells proliferation.** U87 cells were cultured for the indicated periods of time in the absence or in the presence of 10 μg/ml of venom or its fractions (F1-F4). Cell proliferation activity was evaluated by using MTT assay. Measurement of the absorbance was performed at a wavelength of 560 nm. The results are from a representative experiment of two performed in triplicate. *p < 0.05 compared with control (untreated cells).

As shown in Figure 4, F3 treatments slightly inhibited glioma cells growth (of about 15% ± 1.02). F4 had no effect since F4-treated cells were comparable to untreated cells, whereas venom, F1 and F2 were more effective. In particular, *P. noctiluca* venom at the same concentration used for all tested compounds (10 μg/ml) induced a 64% ± 1.06 reduction of cell growth rate after 120 h. F2 caused a consistent growth inhibition of about 45% ± 1.6, whereas the analysis of relative change in proliferation between the control and F1 revealed that it caused the largest reduction of cell survival, approximately 80% ± 2.5. This decrease in cell survival was statistically significant compared with the untreated control (p < 0.05) (Figure 4). Treatment with F1 showed an inhibitory effect on cell proliferation similar to the Treatment of cells with 5 μM Cisplatinum, a chemotherapeutic drug known as potent cytotoxic agent on cancer cell lines, used as a positive control. On the basis of these results we decided to further investigate on the anti-tumor activity of the venom and its active fractions.

### Inhibition of cell adhesion

**Figure 5:**
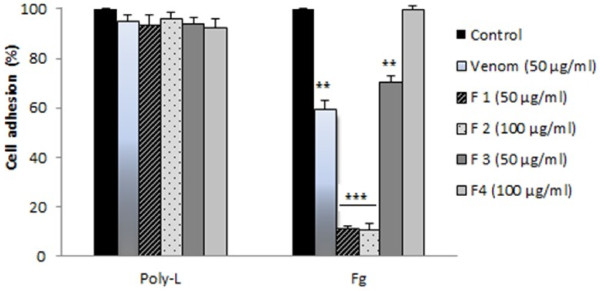
**U87 cells were preincubated without (control) or with 50, 50, 100, 50 and 100 μg/ml of venom, F1, F2, F3 and F4 respectively for 30 min at room temperature.** Cells were then added to 96-well microtiter plates coated with 10 mg/ml with 50 mg/ml fibrinogen (Fg) or poly-L-lysine (Poly-L) and allowed to adhere for 1 h at 37°C. After washing, adherent cells were fixed, stained with crystal violet, solubilized by SDS and the absorbance was measured at 600 nm. **p < 0.01, ***p < 0.001 compared with control (untreated cells).

In a first set of experiments, we have assessed whether *P. noctiluca* venom or its fractions exert an inhibitory effect on adhesion of U87 cells to purified ECM protein used as substratum. Our results clearly showed that the venom at a concentration of 50 μg/ml inhibited attachment of the glioblastoma cell line to fibrinogen, U87 cell adhesion to fibrinogen was also affected by F1, F2 and F3 at a concentration of 50, 100 and 50 μg/ml respectively (concentrations corresponding to IC10). No effect could be observed on the integrin independent substratum, poly-L-lysine, suggesting that the effect of the tested compounds indeed involved the integrin family of adhesion receptors. At a concentration of 100 μg/ml, F4 had no effect on the adhesion of U87 cells neither to fibrinogen nor to poly-L-lysine (Figure 5).

**Figure 6:**
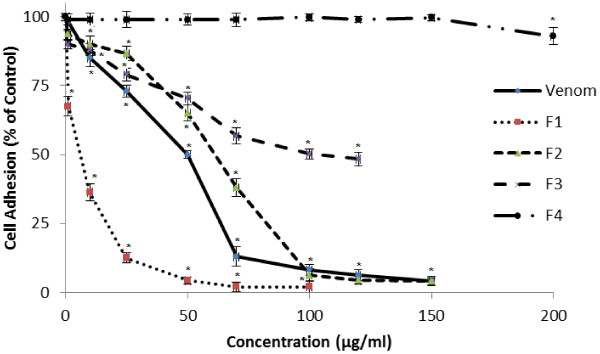
**Dose-effect of*P. noctiluca*venom and its fractions (F1- F4) on U87 cell adhesion to fibrinogen.** N = 4 (at least). Cell adhesion towards fibrinogen was performed in the presence of the indicated concentrations of *P. noctiluca* venom and its fractions (F1- F4). Data shown (± SD) are from one experiment representative of three performed in triplicate. * indicates statistically significant differences (p < 0.05) compared with the control (untreated cells).

In a second set of experiments, we measured the effect of various concentrations of *P. noctiluca* venom or its fractions (F1-F4) against U87 cell adhesion to fibrinogen (Fg). As shown in Figure 6, the venom, F1, F2 and F3 inhibited the adhesion of U87 cells to fibrinogen. This inhibition was dose-dependent, with an IC50 values of 5, 64 and 100 μg/ml respectively. We found that F4 did not significantly affect U87 cells. According to this test, the inhibitory effect of U87 cell adhesion to fibrinogen ranking was F1>venom>F2>F3.

## Discussion

As the toxicity of cnidarian venoms is a well-known subject [25], the aim of this paper is to demonstrate for the first time that the venom of the jellyfish *P. noctiluca*, can also exert anti-tumour activity *in vitro*.

Analysis of *P. noctiluca* venom on SDS-PAGE revealed at least 15 protein bands ranging in molecular weights from 4 to 120 kDa [26]. Sephadex G75 chromatography led to the separation of the venom on four major fractions.

The mechanisms of venom-induced cytotoxicity are particularly interesting mainly for defining the concentration range for further *in vitro* testing [27]. For this purpose we have demonstrated that a decrease in cell viability is moderately induced in U87 cells exposed to crude venom extract of *P. noctiluca* in a dose-dependent manner with an IC50 about 180 μg/ml (Figure 2, Table 1). These results are in accordance with previous findings where *P. noctiluca* venom induced cell mortality on Vero and HCT 116 cell lines [26-28].

After fractionation of *P. noctiluca* venom and screening of pooled fractions for cytotoxic activity, we observed that F1 and F3 caused a market decrease of cell viability in a concentration dependent manner. IC50 values determined after 24 h of cell treatments are 125 μg/ml and 179 μg/ml for F1 and F3, respectively (Table 1). Whereas, F4 and F2 had no cytotoxic effect on U87 cells (Figure 2).

As shown in Figure 3, the treatment of the crude venom extract, F1 and F3 changed the cell morphology of U87 cells from flattened glioblastoma membranous morphology, representing activation state, to slender shape, representing quiescent state (Figure 3 ()). Based on morphological change, we suppose that the U87 cell mortality induced by *P. noctiluca* venom extracts may be achieved by induction of apoptosis but not due to direct toxic effect. To date, *P. noctiluca* venom has been described to have a moderate cytotoxic effects on cancer cell lines and was able to promote apoptotic cell death on Vero cells [28].

One of the most frequent parameters assessed in cancer drug discovery is the impact of a given molecule on the proliferation of a cancer cell [29]. For this reason the effect of the crude venom extract and its fractions on U87 cell proliferation were assessed using MTT assay over a concentration of 10 μg/ml (This concentration is about 17 times less cytotoxic than IC50) after 48, 72, 96 and 120 hours.

Our results has been shown a time-dependent increase of the percentage of growth inhibition. The anti-proliferative activity rates of *P. noctiluca* venom, F1, F2, F3 and F4 were about 60%, 80%, 50%, 20% and 5% respectively after 5 days of exposure (Figure 4). Among these results, crude venom, F1 and F2 appear to be the most interesting fractions and exhibit an antiproliferative effect comparable to that of the drug used clinically for tumor treatment, cisplatinum (5 μM).

Similar to our results, Balamurugan et al. [30] reported the antitumor activity of *Chrysaora quinquecirrha* venom. Data revealed that a peptide with a molecular weight of 9 kDa, isolated from *Chrysaora quinquecirrha* venom by sephadex G-100 column chromatography could induce apoptosis in HEp2 and HeLa cells [30]. This peptide was further studied *in vivo* for its anti cancer activity. Balamurugan et al. reported that nematocyst venom of *Chrysaora quinquecirrha* peptide possessed significant antitumor activity comparable to that of the result obtained from the animals treated with the standard drug 5-fluorouracil [31].

Another study demonstrated that a potent cytolytic proteins and an inhibitor of papain-like cysteine proteinases (equistatin), were isolated from the sea anemone *Actinia equina*[32]. Equistatin has been shown to be a potent inhibitor of papain and an inhibitor of the aspartic proteinase cathepsin D [33]. Papain-like cysteine proteases have been implicated in various diseases of the central nervous system, such as brain tumors [34].

In addition to cell growth, treatment with *P. noctiluca* extracts affected another hallmark of gliomas, i.e. adhesion. According to our knowledge, such inhibitory activities of jellyfish venom on cell adhesion have not been reported yet.

Cancer cells interact with their surrounding cells and matrix proteins in order to replicate, gain nourishment, and migrate to a new location. The interaction of cancer cells with ECM is essential for metastasis, which is the principal cause of death in cancer patients. Cancer cell movement is controlled by various factors produced by cancer cells and host cells, including growth and motility factors, cytokines, cell adhesion molecules and ECM proteins. Cell adhesion molecules play a crucial role in cell motility through cell–cell and cell–ECM interactions [35]. The most-characterized cell adhesion receptors are the integrins. Integrins are cell adhesion molecules composed by the noncovalent association of α and β subunits. Thus, It has notably been shown that several snake venom-derived peptides can affect integrin function. Among these peptides, disintegrins have been widely characterised and are now studied for potential use in medicine [36].

Our findings clearly show that *P. noctiluca* venom inhibited U87 cell adhesion to fibrinogen and had an IC50 of 25 μg/ml. F1, F2 and F3 inhibited cell adhesion to fibrinogen in a dose dependent manner with an IC50 of 5, 64 and 100 μg/ml respectively (Table 1). The inhibition of F1 at 50 μg/ml was 12,5 times more effective when compared to the crude venom extract at the same concentration. F4 was not able to inhibit cell adhesion to fibrinogen. Moreover, no effect could be observed on the integrin independent substratum, poly-L-lysine, suggesting that the effect of *P. noctiluca* extracts indeed involved the integrin family of adhesion receptors (Figure 5). Thus, we hypothesized that crude venom, F1, F2 and F3 could affect the function of this family of adhesion receptors.

Preclinical data indicate that integrins play a key role in cancer initiation and progression [37]. They are primarily responsible for cell adhesion to ECM, and they are thus involved in anchorage-dependent cell proliferation [38,39]. The integrins alpha V beta 3 and alpha V beta 5, among others, are expressed not only on the tumor vasculature and angiogenic endothelial cells, but also on tumor cells, including gliomas (reviewed in [40]). In glioblastoma, overexpression of alpha V beta 3 integrin is well documented [41]. Importantly, immunohistochemistry analysis revealed that alpha V beta 3 integrin expression was mainly confined to the tumor region and was absent in normal tissue [42].

Given the role of integrins in promoting glioma growth, invasion and angiogenesis, integrin inhibitors might be ideal therapeutic tools. Numerous anti-angiogenic components targeting integrin receptors have been isolated from snake venom [43]. These proteins belong to two families, disintegrins and C-type lectin proteins. Since their initial characterization, snake venom disintegrins have been extensively studied [44]. They are potent and specific antagonists of several integrins, such as alpha V beta 3 and alpha 5 beta 1, the disintegrin family was the first to be characterized and the most extensively studied [45]. EMS16, a C-type lectin protein from *Echis multisquamatus* was the first example of a different class of venom proteins showing an antagonistic effect on integrins [46]. In addition, BJcuL, from the snake *Bothrops jararacussu* inhibits tumour and endothelial cell growth [47] and Rhodocetin from *Vipera lebetina venom* antagonises tumour invasion [4].

## Conclusions

In conclusion, we isolated four fractions with sephadex G-75 chromatography from venom of the Mediterranean jellyfish *P. noctiluca* and evaluated their antiproliferative activity separately using U87 cells. Among the four fractions F1 and F2 and F 3 showed significant antiproliferative activity in time-dependent manner. Our results also suggest that these fractions and the crude venom are able to inhibit cell adhesion to fibrinogen in dose- dependent manner. This inhibition is probably reliant on its ability to interact with integrins. Although the specific integrins affected by *P. noctiluca* venom and its active fractions have not been identified in this study, the integrin alpha V beta 3 and alpha 5 beta 1 might be involved. The purification and the determination of chemical structures of compounds of these active fractions are under investigation. Overall, *P. noctiluca* venom may has the potential to serve as a template for future anticancer-drug development. Further analyses are warranted and necessary to substantiate our findings.

## Abbreviations

*P. noctiluca*: *Pelagia noctiluca*; MTT: 3-(4, 5- dimethyl thiazol −2-yl) 2, 5-diphenyl 3-(4,5-dimethylthiazol-2-yl)-2,5-dimethyltetrazolium bromide; IC50: The concentration inducing 50% loss of cell viability; IC10: The concentration inducing 10% loss of cell viability; ECM: Extracellular matrices; Fg: Fibrinogen; poly-L: Poly-L-lysine.

## Competing interests

The authors declare that they have no competing interests

## Authors’ contributions

YA carried out cell cultures, cell proliferation assays and drafted the manuscript. MB carried out cell viability assays and cell adhesion assays. HBM participated to cell cultures and cell adhesion assays. MM performed statistical analysis. NM participated in the design of the study. HB contributed in correcting the manuscript and approved its final version. All authors read and approved the final manuscript.
